# Addressing the Challenges and Opportunities of the Polio Endgame: Lessons for the Future

**DOI:** 10.1093/infdis/jix117

**Published:** 2017-07-01

**Authors:** Manish Patel, Stephen Cochi

**Affiliations:** 1 Centers for Disease Control and Prevention, Atlanta, Georgia

**Keywords:** polio, eradication, poliovirus, endgame, OPV, oral poliovirus vaccine, IPV, inactivated poliovirus vaccine.

## Abstract

The Global Commission for the Certification of the Eradication of Poliomyelitis certified the eradication of type 2 poliovirus in September 2015, making type 2 poliovirus the first human pathogen to be eradicated since smallpox. The eradication of type 2 poliovirus, the absence of detection of type 3 poliovirus worldwide since November 2012, and cornering type 1 poliovirus to only a few geographic areas of 3 countries has enabled implementation of the endgame of polio eradication which calls for a phased withdrawal of oral polio vaccine beginning with the type 2 component, introduction of inactivated poliovirus vaccine, strengthening of routine immunization in countries with extensive polio resources, and initiating activities to transition polio resources, program experience, and lessons learned to other global health initiatives. This supplement focuses on efforts by global partners to successfully launch polio endgame activities to permanently secure and sustain the enormous gains of polio eradication forever.

Polioviruses cause an acute enteric infection that can clinically manifest as acute flaccid paralysis (AFP) and possibly death. In 1988, the World Health Assembly (WHA) formally endorsed efforts to eradicate polio through the Global Polio Eradication Initiative (GPEI). Cases of paralytic polio have declined enormously, from some 350000 cases in 1988 to only 37 cases in 2016 [1]. Paralytic polio is caused by one of 3 wild poliovirus (WPV) types—WPV1, WPV2, and WPV3—each with its unique epidemiology, immune response, and vaccine requirement [2, 3]. All currently remaining cases of WPV infection globally are due to WPV1. The last naturally occurring case of WPV2 infection was in October 1999 and that of WPV3 was in November 2012 [1, 4]. The Global Commission for the Certification of the Eradication of Poliomyelitis certified the eradication of type 2 poliovirus in September 2015, making type 2 poliovirus the first human pathogen to be eradicated since the agent of smallpox [4]. This enormous progress toward polio eradication, including the absence of type 3 for >4 years and the restriction of WPV1 to only a few high-risk states of 3 countries, brings the eradication efforts to its final chapter, otherwise known as the polio endgame. Reasons for the success of polio eradication efforts to date include advances in polio research and innovation, vaccinology, laboratory detection, and genotyping; epidemiology; policy; and intense and innovative efforts to deliver vaccines to target populations worldwide [5–13]. This supplement of *The Journal of Infectious Diseases* focuses on the operationalization of efforts to ensure a successful polio endgame, including essential changes in global polio vaccination policy, namely, the beginning of phased withdrawal of oral poliovirus vaccine (OPV), combined with the introduction of inactivated poliovirus vaccine (IPV), and the launching of activities to document the transition of polio resources, lessons, and learnings to other priority global health initiatives (Figure 1).

**Figure 1.**
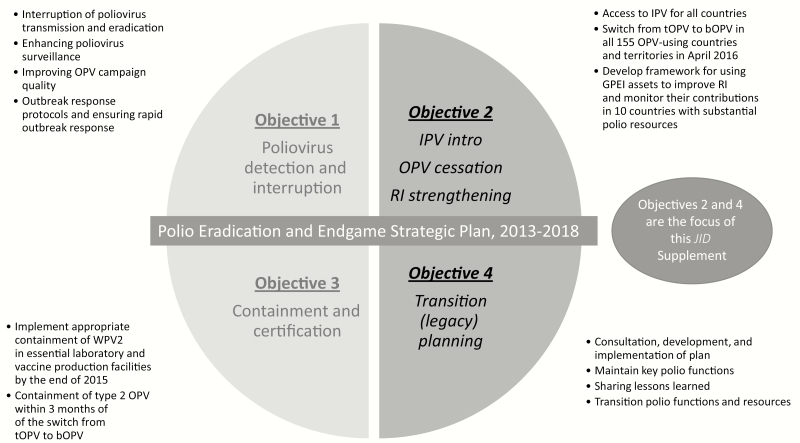
The Polio Eradication and Endgame Strategic Plan, 2013–2018. Abbreviations: bOPV, bivalent oral poliovirus vaccine; GPEI, Global Polio Eradication Initiative; IPV, inactivated poliovirus vaccine; OPV, oral poliovirus vaccine; RI, routine immunization; tOPV, trivalent oral poliovirus vaccine; WPV2, type 2 wild poliovirus.

Historically, vaccinologists have developed and endorsed 2 vaccines (trivalent OPV and IPV) for the control, elimination, and anticipated eradication of polio [14, 15]. Each vaccine contains all 3 polioviruses, either as live attenuated viruses administered orally (for OPV) or inactivated viruses administered thru parenteral injections (for IPV). Experts worldwide have intensely debated the role of these vaccines in eradication efforts over the years [16–27]. An understanding of these vaccines has also evolved over the years, and, although both vaccines have been in use for >50 years, a global consensus about the joint role of the 2 vaccines in polio eradication efforts has only recently taken shape [5, 28–30].

Over the years, the polio endgame strategy has also evolved. At the outset, the envisioned strategy was simpler: to eradicate the world of WPVs through the use of OPV, followed by discontinuation of OPV [31–33]. However, on the basis of an improved understanding of polioviruses and vaccines, current eradication efforts necessitate a more nuanced and, undoubtedly, complicated approach to the endgame [34–38]. The use of OPV is now accepted to be a double-edged sword: while OPV has been the primary reason for the enormous success of polio control globally, it also, on very rare occasions, causes either sporadic vaccine-associated paralytic polio (VAPP) in vaccine recipients or close contacts or outbreaks of vaccine-derived polioviruses (VDPVs). In the latter instance, during gut replication and transmission in subsequent chains of contact, OPV evolves through multiple genetic nucleotide changes and frequently recombination with nonpoliovirus enterovirus C species [39]. In very rare cases, the vaccine virus may regain neurovirulence and acquire transmissibility comparable to that of WPV, thus causing outbreaks of paralytic polio related to VDPVs that are clinically indistinguishable from paralysis due to WPVs. Dozens of similar outbreaks have been reported worldwide since the first well-documented, confirmed outbreak of VDPV in Hispaniola, in 2000 [10, 39, 40]. Most of these outbreaks have occurred in areas with low OPV coverage, which creates a large pool of polio-susceptible persons and allows VDPVs to circulate (cVDPVs). However, in no uncertain terms, cases of paralysis due to cVDPV are very rare and far outweighed by the immense benefits of OPV.

As WPVs are eradicated, the world must cease the use of OPV because the global eradication of polio ultimately must include the eradication of paralytic polio due to all forms of live polioviruses. The recurrent detections of cVDPV outbreaks prompted experts to rethink the endgame strategy [30, 34, 36, 41]. WPV2 has been eradicated, but type 2 virus accounted for approximately 90% of all reported cVDPVs between 2000 and 2014 and approximately 26%–31% of vaccine-associated paralytic polio cases [41, 42]. Furthermore, type 2 vaccine virus is the most immunogenic of the 3 vaccine virus strains in OPV and interferes with the replication of types 1 and 3 in the intestinal tract and, hence, their “take” or immune response. Thus, OPV withdrawal would have to be phased beginning with cessation of the type 2 component of OPV (OPV2) through a switch from the use of trivalent OPV (tOPV contains poliovirus types 1, 2, and 3) to the use of bivalent OPV (bOPV contains only poliovirus types 1 and 3) [43]. Use of bOPV would continue until certification of eradication of WPV1 and WPV3.

Cessation of OPV2 use has the inherent risk of reemergence of type 2 virus (Figure 2) [43, 44]. Models have shown that the risk of reemergence of cVDPV2, although very low, would increase during the first 1–2 years after OPV2 cessation, owing to declining type 2 immunity in the context of silent or undetected transmission of cVDPV2 [45–47]. Longer-term risk of WPV may also exist if a breach in laboratory containment or intentional release of virus occurred [48, 49]. The GPEI’s approach was to mitigate these risks through a series of key readiness steps prior to the switch, including controlling cVDPV2 outbreaks, ensuring high immunity to type 2 poliovirus, preparing a postswitch type 2 surveillance and outbreak response protocol, expanding environmental surveillance, stockpiling monovalent OPV2 to deal with potential outbreaks, and containing WPV2 to essential facilities [30, 35, 41, 43, 50–55]. Perhaps most relevant to the world was that 126 countries worldwide would have to introduce IPV into their routine immunization (RI) system to help reduce the risks of reintroducing type 2 poliovirus by providing some level of population immunity against type 2 and facilitating interruption of transmission if outbreaks occur [43]. Fundamentally, all countries would need a convincing rationale, clear policy and mandate, and sufficient resources to use IPV, which is costlier and logistically more difficult to administer than OPV but does not cause paralytic polio. Implementation of this part of the polio endgame had all the makings of a complex endeavor.

**Figure 2.**
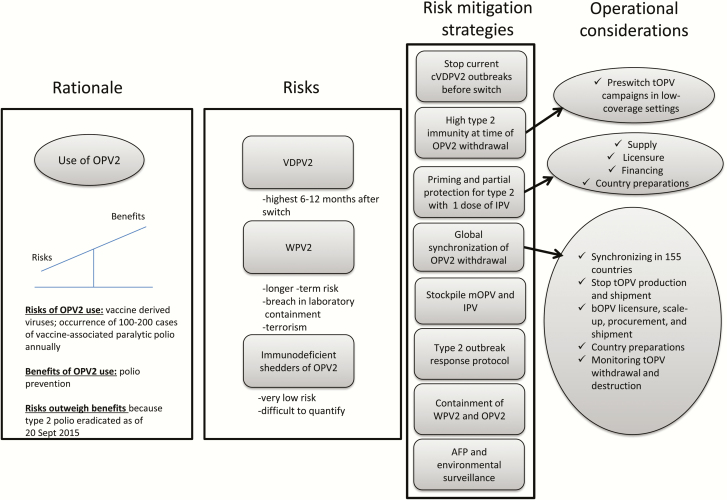
Rationale, risks, mitigation strategies, and operational considerations for withdrawal of the type 2 component of oral poliovirus vaccine (OPV2). Abbreviations: AFP, acute flaccid paralysis; bOPV, bivalent oral poliovirus vaccine; cVDPV, type 2 circulating vaccine-derived poliovirus; IPV, inactivated poliovirus vaccine; mOPV, monovalent oral poliovirus vaccine; tOPV, trivalent oral poliovirus vaccine; WPV2, type 2 wild poliovirus.

Polio eradication efforts are resource intensive and cannot go on forever. Once transmission of wild viruses has been interrupted and eradication certified, a narrow window of time exists within which the endgame must be accomplished. In anticipation of eradication of type 2 poliovirus, the WHA in May 2013 endorsed the GPEI’s Polio Eradication and Endgame Strategic Plan, 2013–2018 [37]. The plan outlines 4 objectives (Figure 1) that comprehensively address eradication of polio, the endgame strategy, containment of polioviruses to essential facilities, and the polio legacy (ie, transition) process. Objective 2 deals with the endgame component calling for the phased withdrawal of OPV, beginning with the withdrawal of the type 2 component in 2016, and objective 4 calls for initiating actions to maintain and mainstream essential polio functions (eg, surveillance, immunization, and containment) in a post–polio eradication world and transition the investment in polio eradication to benefit other priority public health initiatives for years to come [41, 56]. Between 2013 and 2016, all 126 countries using OPV would require access to at least 1 dose of IPV, and efficient use of polio resources would need to occur in the 10 priority countries where the GPEI presence is strong and most of the polio infrastructure is present, to strengthen their RI services and facilitate OPV2 withdrawal in 2016. During 2 weeks in April 2016, all 155 OPV-using countries and territories would have to withdraw OPV2 through a globally synchronized switch from the use of tOPV to bOPV. Implementation of the various aspects of objectives 2 and 4 of the endgame plan are covered by articles in this supplement, through 4 sections: IPV, switch, strengthening immunization services, and transition planning. The supplement is not a comprehensive representation of all work in the global arena related to the polio endgame. Rather, it provides a glimpse of key activities within the key domains of the endgame across global, regional, and national levels.

## COORDINATION OF THE ENDGAME

To implement the endgame, in 2014 GPEI launched the Immunization Systems Management Group, which consisted of members from the 5 core partner agencies and Gavi, the Vaccine Alliance. The 5 agencies—the World Health Organization (WHO), the United Nations Children’s Fund (UNICEF), the Centers for Disease Control and Prevention (CDC), Rotary, and the Bill and Melinda Gates Foundation—are the core partners of the GPEI but also happen to be some of the most influential and heavily involved agencies in the global immunization arena. Implementation of the endgame was a result of a truly successful collaboration among these large agencies with complex working structures and bureaucracies and can serve as a model for future collaborations associated with other similarly challenging global health initiatives. The first section of the supplement deals with a series of articles that describe the lessons learned by the global partners with regard to the structure of the global coordinating mechanism [57], the financial support mechanisms [58, 59], regulatory challenges [60], communications and advocacy efforts [61], supply considerations [62, 63], and global coordination of implementing the polio endgame [64].

## IPV INTRODUCTION

While coordination and support from the global level was important, implementation ultimately occurred at the national level with support from regional offices of the WHO and UNICEF and local implementation partners. Colleagues from the regional offices discussed the challenges and creative solutions implemented at the regional and national levels when introducing IPV [65–69]. Ba-Nguz et al describe country level efforts to engage national immunization advisory groups to facilitate country-specific policy considerations in Indonesia and Uganda [70]. Scotney et al cover the broad spectrum of challenges and lessons with IPV introduction in Cameroon, Nigeria, and Kenya [71]. Evaluations from Bangladesh illustrate implementation challenges related to ensuring cold chain capacity, minimizing wastage, acceptability of vaccine, and improving coverage [72, 73]. The introduction of IPV into RI programs that already used pentavalent vaccine (which covers diphtheria, tetanus, pertussis, *Haemophilus influenzae* type b, and hepatitis B) and pneumococcal vaccine resulted in the need to administer 3 injections during a single visit. Concerns emerged from staff of national immunization programs and providers from all regions of the world with regard to the safety and acceptability of multiple injections. Dolan et al and Subaiya et al provide evidence from a global landscape analysis and a recent evaluation from Albania that support the notion that caretakers and providers appreciate the health benefits of vaccines and deem them to outweigh the concerns of pain or inconvenience associated with multiple injections [74, 75]. These issues were evaluated by the WHO’s Strategic Advisory Group of Experts (SAGE) on Immunization, which concluded that delivery of pentavalent vaccine, pneumococcal vaccine, and IPV at a single visit was safe and effective, as well as acceptable to vaccinators and caregivers in many countries [76].

## OPV2 CESSATION: SWITCHING FROM TRIVALENT TO BIVALENT OPV

Between mid-April and mid-May 2016, all 155 OPV-using countries and territories had discontinued use of type 2 OPV by switching from tOPV to bOPV in their national immunization programs. The switch included a complex milieu of activities, including cessation of tOPV production and shipment by manufacturers, national inventories of tOPV, detailed forecasting of tOPV needs, bOPV licensing, scaling up of bOPV production and procurement, developing national operational switch plans, securing funding, establishing oversight and implementation committees and teams, training logisticians and health workers, fostering advocacy and communications, establishing monitoring and validation structures, and implementing waste management strategies (Figure 2). These activities had to occur across global and regional levels and involved a complex interplay between various agencies, national governments, and manufacturers. Most importantly, the switch had to be synchronized in 155 countries and territories with varying levels of infrastructure, capacity, legislation, and manufacturer contracts [43]. This unprecedented achievement was in no uncertain terms related to a strong collaborative partnership between national governments, United Nations agencies, supporting partner agencies, donors, regulatory agencies, and manufacturers.

Several articles illuminate factors leading to a successful switch, including advance planning, establishment of policies designed to protect populations while ensuring sufficient programmatic flexibility to safely implement the switch, identification and dissemination of human and financial resources, and timely dissemination of standardized and easily digestible protocols. Supply considerations were the lynchpin of the synchronized withdrawal, to ensure that sufficient tOPV was available until the switch without an abundance of excess unused stocks that would require destruction after the switch. Further complicating the matter, countries have varying electronic and paper-based stock management systems, contracts, suppliers, stocks, capacity for training and monitoring, and legislations and resources for vaccine disposal.

Rubin et al describe the global vaccine supply market’s supply considerations for the planned cessation of tOPV use [62]. Ramirez et al provide intricate details on the overall switch planning and implementation from a global perspective, while several articles by colleagues from WHO and UNICEF regional offices provide regional and national experiences with the switch [77]. Although planning, strategy, and coordination across all levels (global, national, and local) among relevant partners and stakeholders is crucial, monitoring outcomes provides greater confidence regarding the validity of the OPV2 withdrawal. With some 2 billion doses of tOPV in use each year across several hundred thousand health facilities in 155 countries and territories, the sheer magnitude of the task of monitoring and validating the withdrawal was unwieldy. However, Farrell et al describe a practical and sound monitoring and validation strategy that resulted in 99%, 95%, 77%, and 24% of vaccine stores at national, regional, district, and health facility levels, respectively, being monitored globally in a short span of 2 weeks to ensure that the vast majority of the tOPV was withdrawn from use during the globally synchronized switch window [78]. Because withdrawn tOPV could still make its way back to the cold chain, possibly resulting in ongoing use of OPV2 and generation of VDPVs, destroying all withdrawn tOPV was a crucial aspect of the switch. Wanyoike et al provide an overview of challenges and novel, practical solutions for destruction and disposal of unused vaccines simultaneously across all countries, particularly in resource-poor settings [79]. Destruction of unused vaccines largely has been an unchartered territory prior to the switch, and documenting these experiences provides relevant learnings for the withdrawal of all OPV after eradication.

## STRENGTHENING IMMUNIZATION SERVICES

For eradication to succeed, immunization systems must be capable of adopting and delivering vaccines according to the national immunization schedules vis-à-vis the RI system. Increasing polio immunity by vaccines provided through the RI system is one of the important pillars of polio eradication. Moreover, a robust RI system also provides the foundation for controlling all childhood vaccine preventable diseases. Thus, efforts to eradicate polio are not occurring in a vacuum. Polio resources—finances, personnel, equipment, and experience—contribute to RI services particularly in countries with a large GPEI presence. The endgame activities offered unique opportunities to improve collaborations between global immunization partners working on polio and on RI systems and make efficient use of GPEI resources to strengthen RI activities. This was particularly relevant for countries with a large GPEI presence, high-risk target populations, and weak immunization systems.

Several of the articles in this supplement take stock of how GPEI resources support RI activities and contribute to strengthening RI services, with the specific aim of identifying services that may be at risk when polio resources diminish after eradication [80, 81]. Deutsch et al and Ongwae et al describe how use of polio resources, social mobilization networks, and experience may also be valuable to broader health programs and RI activities [82, 83]. Van den Ent et al identify several key factors behind the success of polio eradication programs in the 10 high-risk priority countries, including government leadership, evidence-based programing, community partnership, and strong accountability systems [84]. They provide specific examples of how these factors and experiences have been positively leveraged to broader immunization activities and provide motivation for pursuing synergies between polio and RI programs. With such efforts, both horizontal and vertical immunization programs can indeed coexist synergistically as they strive to meet their shared goal of improving child health globally.

## TRANSITION PLANNING

As illustrated by Van den Ent et al, the polio infrastructure, workforce, and financial resources substantially contribute to immunization services beyond polio [81]. Moreover, the GPEI has amassed tremendously useful lessons and programmatic experience during the past 3 decades in what has been one of the largest mobilizations of the public health community in history toward any one disease. To secure the gains of polio eradication for future generations requires that some essential functions of the polio program will need to continue to maintain immunity after polio eradication has been certified, to sustain acute flaccid paralysis and environment surveillance, and to maintain outbreak response capacity. Rutter et al provide an overview of post–polio eradication transition planning and describe both the considerable risks associated with the loss of current polio assets and infrastructure, as well as the substantial opportunities to build on these investments to benefit other national and global health priorities [85]. Transition planning (previously referred to as “legacy”) has been a core component of the endgame plan, to continue the forward progress of global and national immunization programs in the face of the anticipated ramp down of polio resources and infrastructure that currently contribute substantially toward supporting overall ongoing immunization systems. The challenge is to effectively and responsibly transition polio resources, capacities, and experiences toward other global public health priorities [56].

The 2016 mid-term review of the Global Vaccine Action Plan emphasized that “all countries should mitigate any risk to sustaining effective immunization programs when polio funding decreases” [86, p. 21]. Several articles highlight how the capacities and assets of the GPEI have been supporting other immunization and global health priorities and how this support must continue or risk negative consequences to immunization and health systems. Williams et al describe how the polio surveillance system serves as a platform for vaccine-preventable disease surveillance [87]. The associated support, experience, and lessons learned have played a key role in the development of the global and regional networks for vaccine-preventable diseases [88, 89]. The GPEI has contributed substantially to efforts to eliminate measles and rubella globally and nationally [90, 91]. GPEI-initiated social mobilization networks and polio-funded global positioning system mapping activities to support microplanning have had their use extended to other health priorities [92, 93]. The Stop Transmission of Polio program has expanded its workforce development activities far beyond polio to now include RI activities, vaccine-preventable disease surveillance, measles and rubella elimination, communications and social mobilization, and immunization data quality [94, 95]. These are just a few examples of the reach of the GPEI and what is at stake if the potential opportunities of polio transition planning are not meaningfully addressed.

## KEY FUTURE CHALLENGES

Although the world has made substantial progress with the polio endgame during the past 3–4 years, the articles in the supplement also highlight specific challenges and gaps that are relevant for future immunization work. These include areas such as capacity building through national immunization advisory groups, national regulatory mechanisms, self-procurement of vaccines, financing and supporting immunization activities in middle-income countries, expanding and maintaining a functioning cold chain, stock management and transport, waste management, and issues related to acceptability of multiple injections. These all tie in to the contention that so-called vertical programs, such as polio eradication or the introduction of new vaccines, may rely on a strong RI system for success but may not necessarily strengthen such systems. The strengthening of RI systems deserves to have its own investment and attention.

Ultimately the countries are to be commended for introducing or committing to introduce at least 1 dose of IPV prior to the switch. However, the 2 manufacturers who had committed to meet the demand of the planned accelerated IPV introduction have encountered repeated unforeseen setbacks in providing the contractually agreed upon IPV supply related to the rapid scale up of complex production processes. Thus, many of the countries are currently facing shortages or stockouts of IPV [96]. However, global partners, manufacturers, and countries are working closely to find innovative and acceptable solutions for this temporary setback, including apportioning the limited stocks to the highest-risk populations and using dose-sparing fractional doses (ie, one fifth of the full vaccine dose [fIPV]) of IPV in willing countries. Hiro et al provide a review of the immunogenicity and operational data supporting intradermal use of fIPV [97]. The use of fIPV in RI activities and, potentially, in supplementary immunization activities or outbreaks could ease the supply situation some but would require logistical considerations for countries, such as high wastage with 10-dose vials, potential regulatory concerns from off-label use of fIPV, challenges of procuring intradermal syringes or devices, and training health workers in administrating vaccine intradermally. India and Sri Lanka have successfully adopted 2 doses of fIPV in the RI system nationwide [96, 98]. Others may follow their lead, although countries have expressed concern about logistical challenges, competing priorities, and assurances of supply even with fIPV. Global partners for years have also invested in a long-term strategy of engaging additional suppliers and immunization strategies that would provide protection against polio after eradication, including Sabin IPV, adjuvanted IPV, combination IPV products, safer versions of OPV, and microneedle patches [30].

The switch from tOPV to bOPV has been largely deemed a success. With some 2 billion doses used per year globally, the goal of the globally synchronized switch was to mitigate risk by reducing risk as much as possible while balancing risk with other priorities and available resources. Since the switch, environmental and stool surveillance has identified sporadic detections of Sabin-like type 2 polioviruses, indicating some ongoing use of tOPV after the switch. After these detections in Hyderabad and Ahmedabad, India, an extensive investigation 4 months after the switch identified some very limited use of tOPV in a few private clinics (approximately 50 vials in 29 of approximately 5000 private clinics or retailers sampled) [99]. Thus, countries were unlikely to have withdrawn every single vial, particularly from the private sector, which was challenging to monitor by most governments. Some isolated scenarios of use of limited amounts of tOPV are not surprising given the magnitude of tOPV use each year prior to the switch. However, in the first year after the switch, no outbreaks of cVDPV2 have been attributed to ongoing use of tOPV, supporting the contention that the vast majority of tOPV has been withdrawn globally. Hampton et al discuss implications of the switch experience for all OPV withdrawal after polio eradication [100]. They note that the higher stakes after polio eradication may justify a more aggressive approach for all OPV withdrawal, involving advanced planning, funding, monitoring, and attention to withdrawal from the private sector.

## CONCLUSION

The outcomes of introducing IPV in nearly all countries in an accelerated manner in a compressed time frame of 3 years and successfully withdrawing OPV2 globally during April–May 2016 supports the view that the Immunization Systems Management Group, stakeholders, and national governments and immunization programs succeeded in achieving the first phase of the polio endgame. Moreover, they did so without seriously affecting other day-to-day operations, including introductions of new vaccines or responses to outbreaks. For example, during this period 19 countries introduced rotavirus vaccine, and 16 introduced pneumococcal vaccine [101]. Many of the countries also dealt with serious civil or regional conflicts and outbreaks (such as those due to Middle East respiratory syndrome coronavirus, Ebola virus, and Zika virus). The collective experience outlined in the articles in this supplement provides optimism for future global health initiatives, such as measles and rubella eradication. From the endgame experience, key factors for success include a global mandate from country authorities (eg, the WHA), clarity in policy (eg, Strategic Advisory Group on Immunization guidance), financial resources (eg, donor support), and commitment from the highest levels of core partner agencies. Moreover, the articles also indicate that the alignment of these global factors for the polio endgame fostered and facilitated a true collaborative spirit among highly motivated individual members of the partner agencies who are committed to the goal of polio eradication. The articles authored by these individuals in this supplement serve as an optimistic example of what is achievable in global health through effective collaboration and determination. They also provide a blueprint for future work by the GPEI as it closes in on polio eradication and withdraws all OPV globally in the near future, thus securing the gains of eradication permanently.

All in all, the work summarized in this supplement demonstrates that, with proper support, country immunization programs have a strong capacity to absorb and adapt to new global health initiatives in immunization, which are ultimately designed to reduce the burden of infectious diseases and provide healthier lives for children worldwide. Eradication of a disease from the human population is not easy, which is why it has only been accomplished once in human history. However, the permanency of eradication justifies the effort put forth to provide the ultimate form of equity in health, both for those living today and for future generations to come.

## Notes

***Financial support.*** This work was supported by the Bill and Melinda Gates Foundation (grant OPP1095024 to M. P.).

***Supplement sponsorship.*** This work is part of a supplement coordinated by the Task Force for Global Health with funding provided by The Bill and Melinda Gates Foundation and the Centers for Disease Control and Prevention.

***Potential conflicts of interest.*** Both authors: No reported conflicts. Both authors have submitted the ICMJE Form for Disclosure of Potential Conflicts of Interest. Conflicts that the editors consider relevant to the content of the manuscript have been disclosed.
